# The NLRP3 inflammasome is essential for IL-18 production in a murine model of macrophage activation syndrome

**DOI:** 10.1242/dmm.050762

**Published:** 2024-07-30

**Authors:** Tara A. Gleeson, Christina Kaiser, Catherine B. Lawrence, David Brough, Stuart M. Allan, Jack P. Green

**Affiliations:** ^1^ Division of Neuroscience, School of Biological Sciences, Faculty of Biology, Medicine and Health, University of Manchester, Manchester M13 9PT, UK; ^2^ Geoffrey Jefferson Brain Research Centre, The Manchester Academic Health Science Centre, Northern Care Alliance NHS Foundation Trust, University of Manchester, Manchester M6 8HD, UK; ^3^ Lydia Becker Institute of Immunology and Inflammation, University of Manchester, Manchester M13 9PL, UK; ^4^ Swedish Orphan Biovitrum (Sobi), AB, Stockholm 112 76, Sweden

**Keywords:** Cytokine storm syndrome, Hyperinflammation, IL-18, Inflammasome, NLRP3

## Abstract

Hyperinflammatory disease is associated with an aberrant immune response resulting in cytokine storm. One such instance of hyperinflammatory disease is known as macrophage activation syndrome (MAS). The pathology of MAS can be characterised by significantly elevated serum levels of interleukin-18 (IL-18) and interferon gamma (IFNγ). Given the role for IL-18 in MAS, we sought to establish the role of inflammasomes in the disease process. Using a murine model of CpG-oligonucleotide-induced MAS, we discovered that the expression of the NLRP3 inflammasome was increased and correlated with IL-18 production. Inhibition of the NLRP3 inflammasome or the downstream caspase-1 prevented MAS-mediated upregulation of IL-18 in the plasma but, interestingly, did not alleviate key features of hyperinflammatory disease including hyperferritinaemia and splenomegaly. Furthermore blockade of IL-1 receptor with its antagonist IL-1Ra did not prevent the development of CpG-induced MAS, despite being clinically effective in the treatment of MAS. These data demonstrate that, during the development of MAS, the NLRP3 inflammasome was essential for the elevation in plasma IL-18 – a key cytokine in clinical cases of MAS – but was not a driving factor in the pathogenesis of CpG-induced MAS.

## Teaser

**Summary:** Interleukin-18 is a key cytokine in clinical cases of macrophage activation syndrome (MAS). Inhibition of the NLRP3 inflammasome reduces plasma levels of interleukin-18 but does not alleviate clinical features of MAS in mice.

## INTRODUCTION

Cytokine storm syndromes (CSS) encompass a variety of disorders that present with hyperinflammation and multi-organ dysfunction characterised by excessive release of cytokines (hypercytokinaemia) (Fajgenbaum and June, 2020). CSS are generally defined by the underlying inflammation driving the cytokine storm response, including infections (Ragab et al., 2020), rheumatic diseases – such as systemic juvenile idiopathic arthritis (SJIA) (Ravelli et al., 2016), and adult-onset Still disease (AOSD) (Iwamoto, 2007), systemic lupus erythematosus (SLE) (Liu et al., 2018), malignancy (Turnquist et al., 2020), immunotherapy (Sandler et al., 2020; Abdelkefi et al., 2009) or genetic defects, such as primary haemophagocytic lymphohistiocytosis (pHLH) (Filipovich and Chandrakasan, 2015; Schulert and Cron, 2020). CSS is commonly known as secondary hemophagocytic lymphohistiocytosis (sHLH) when occurring secondary to malignancy or infection but, more specifically, as macrophage activation syndrome (MAS) in rheumatic disorders (Crayne et al., 2019). Crucially, CSS can be lethal if left untreated, highlighting the need to understand the biology behind CSS to provide effective therapies.

The characteristics of the cytokine storm depend on the causative factor; however, in the case of MAS, several cytokines have been proposed to contribute towards cytokine storm development, with the inflammasome-derived cytokines interleukin-1 beta (IL-1β) and interleukin-18 (IL-18) being implicated in disease pathogenesis (Takada et al., 1999; Krei et al., 2021). IL-1β and IL-18 are produced as precursor proteins, and are cleaved by caspase-1 (CASP1) to generate biologically active forms, with caspase-1 being activated by inflammasomes (Dinarello, 1996; van de Veerdonk et al., 2011). Inflammasomes are multimolecular protein complexes containing sensor pattern recognition receptors (PRRs), such as NLRP3, NLRC4, AIM2 and NLRP1, the adaptor protein apoptosis-associated speck-like protein containing a CARD (PYCARD, also known as ASC), and the protease caspase-1 (Broz and Dixit, 2016; Martinon et al., 2002; Schroder and Tschopp, 2010). Upon inflammasome formation caspase-1 is recruited, leading to auto-proteolytic activation. This triggers to two distinct events: 1) cleavage of the inactive IL-1β and IL-18 precursors (pro-IL-1β and pro-IL-18, respectively) to their bioactive forms and, 2) cleavage of the pore-forming protein gasdermin-D (GSDMD), which allows for release of mature IL-1β and IL-18 (Latz et al., 2013). The subsequent plasma membrane rupture is mediated by clustering of the transmembrane protein NINJ1, which leads to the highly inflammatory form of cell death, known as pyroptosis (Kayagaki et al., 2021). Both IL-1β and IL-18 signalling is intrinsically controlled by the IL-1 receptor antagonist (IL1RN, hereafter referred to as IL-1Ra) and the IL-18 binding protein (IL18BP, hereafter referred to as IL-18BP), respectively (Seckinger et al., 1987; Novick et al., 1999).

Current understanding of MAS highlights two primary cytokines that are involved in the pathogenesis of the cytokine storm: interferon gamma (IFNγ) and IL-18. Furthermore, IL-18 is understood to be a key driver of IFNγ production, indicating that these cytokines are partaking in a feedback loop (Nakamura et al., 1989; Okamura et al., 1995). Clinically, IL-18 is used as an important marker of MAS (Weiss et al., 2018; Shimizu et al., 2010; Mazodier et al., 2005). Blood circulation levels of IL-18 are elevated in both patients with SJIA and AOSD (Shimizu et al., 2013; Jelusic et al., 2007; Girard et al., 2016), and significantly increased during episodes of MAS (Shiga et al., 2021; Krei et al., 2021); this significant elevation in IL-18 diagnostically distinguishes MAS flares from underlying rheumatic disease (Weiss et al., 2018). Mouse models of MAS/HLH also present with increased levels of IL-18 (Weiss et al., 2018; Girard-Guyonvarc'h et al., 2018) and development of MAS is worse in IL-18BP knockout mice (Girard-Guyonvarc'h et al., 2018). Treatment with recombinant IL-18BP (also known as tadekinig alfa) has been shown to reduce symptoms of MAS in AOSD patients (Gabay et al., 2018). Further, roles have also been proposed for IL-1 cytokines in MAS pathogenesis because IL-1Ra is currently used off-label for the treatment of CSS, with patients responding well to high doses (Miettunen et al., 2011; Bruck et al., 2011; Phadke et al., 2021). Mouse models of hyperinflammatory disease also demonstrate an IL-18 signature in the blood. One MAS mouse model in particular is induced by repeated administration of CpG oligonucleotide (ODN) comprising unmethylated CpG dinucleotides (hereafter referred to as CpG) that bind the mouse Toll-like receptor 9 (TLR9). In these mice, a phenotype similar to that observed in patients with MAS is induced (Behrens et al., 2011). Other models of hyperinflammatory disease rely more on IFNγ signalling rather than IL-18 signalling, more closely resembling pHLH (Jordan et al., 2004; Sepulveda et al., 2013). The CpG-induced MAS model has been used to uncover the dynamics of IL-18 and/or IFNγ signalling in disease (Girard-Guyonvarc'h et al., 2018; Gao et al., 2021; Canna et al., 2013). Despite evidence for the involvement of the cytokines IL-1 and IL-18 in MAS pathogenesis, the mechanisms driving MAS remain unclear. Presently, the NLRC4 inflammasome has been implicated in one instance of MAS, known as NLRC4-MAS, where gain-of-function mutations in NLRC4 drive MAS pathogenesis (Canna et al., 2014, 2017; Chear et al., 2020). However, the mechanisms promoting other instances of CSS and MAS remain unclear, and the role of inflammasomes in these syndromes has yet to be fully elucidated.

In this study, we investigated the role of inflammasomes and IL-1 cytokines in hyperinflammation by using a mouse model of CpG-induced MAS. Here, we show that the NLRP3 inflammasome is upregulated during the development of MAS, with tissues displaying elevated levels of NLRP3, caspase-1 and IL-18 following induction of hyperinflammation. However, pharmacological inhibition of the NLRP3 inflammasome, caspase-1 or the IL-1 receptor did not prevent development of MAS symptoms, despite a reduction in plasma IL-18 levels following inhibition of NLRP3 or caspase-1. Our data suggest that, whilst the NLRP3 inflammasome is responsible for increased blood circulation levels of IL-18 in MAS, it is not responsible for the development of other features of MAS pathogenesis in CpG-induced MAS, such as hyperferritinaemia and splenomegaly, and therefore, indicate that alternative mechanisms are responsible for the splenomegaly, hypercytokinaemia and organ dysfunction in MAS.

## RESULTS

### Repeated TLR9 stimulation results in the initiation of hyperinflammatory disease and upregulates the inflammasome

**Fig. 1.**
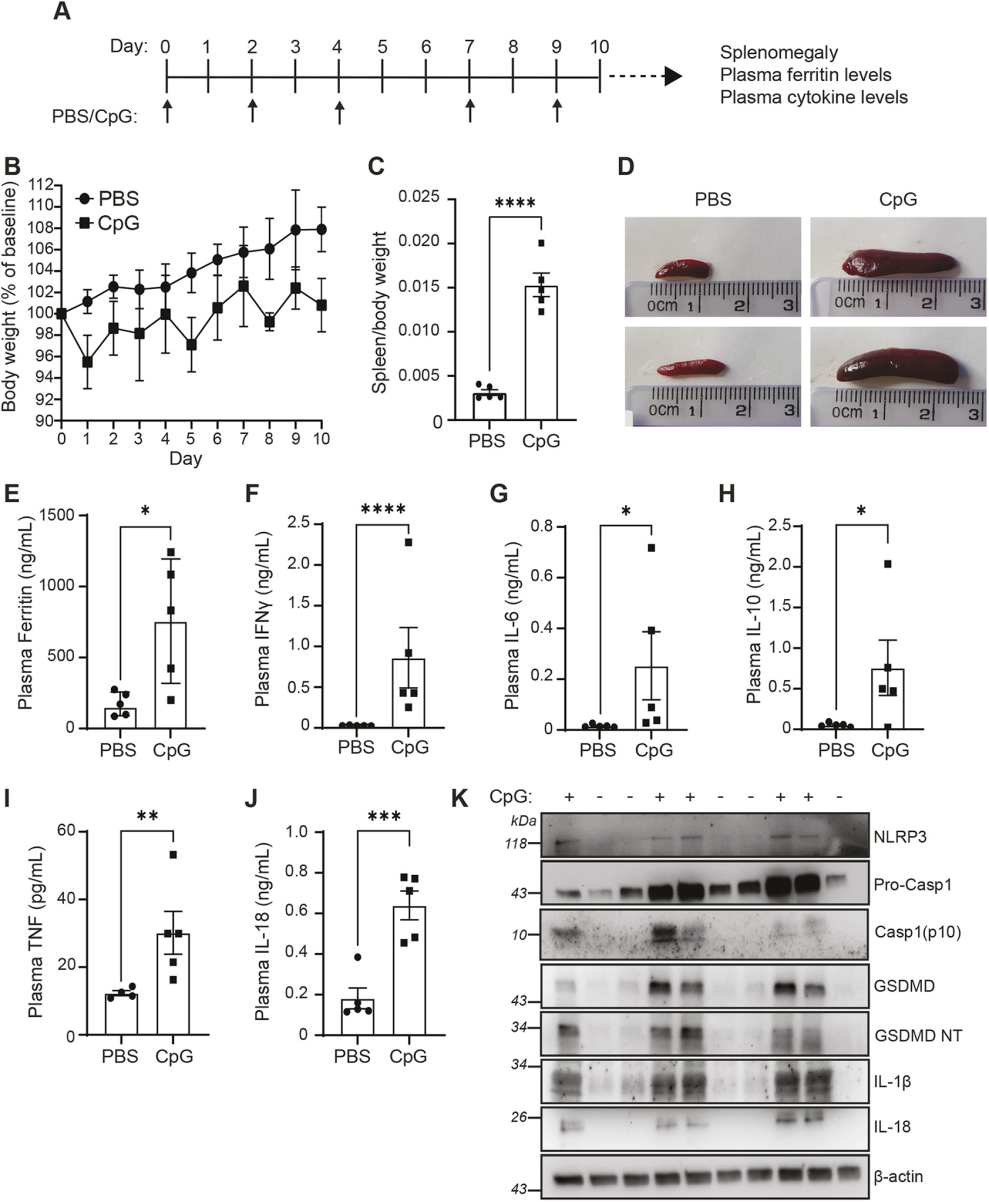
**Repeated administration of CpG induces characteristics of hyperinflammatory disease and inflammasome signalling.** (A) Mice were treated over a 10-day period with CpG (2 mg kg^−1^) or vehicle (PBS), by intraperitoneal injection on days 0, 2, 4, 7 and 9, and sacrificed on day 10 to record hyperinflammatory readouts. (B) Animal weight was measured daily over the course of the study. Animal weights were plotted as a percentage of weight on day 0. (C) Splenic weight normalised to body weight of CpG- or PBS-injected mice (*n*=5). (D) Representative images of spleens obtained from mice as described in C. (E) Plasma levels of ferritin (*n*=5). (F-J) Plasma concentrations of IFNγ (F), IL-6 (G), IL-10 (H) and TNF (I), IL-18 (J) in mice injected as defined in A (*n*=5). (K) Western blot of homogenised spleens from animals treated with PBS (–) or CpG (+) blotted for inflammasome components NLRP3, pro-caspase-1 (Pro-Casp1), caspase-1(p10), GSDMD, cleaved GSDMD N-terminal (GSDMD NT), IL-1β and IL-18 (*n*=5). Data represent the mean±s.e.m. *P<0.05, **P<0.01, ***P<0.001, ****P<0.0001 (unpaired Student's *t*-test)*.*

We used a previously described murine model of MAS, in which repeated intraperitoneal administration of the CpG-oligonicleotide 1826 (ODN 1826, hereafter referred to as CpG), which acts as a TLR9 agonist, induces features of hyperinflammatory disease, similar to those observed in patients with MAS (Behrens et al., 2011). We administered CpG (2 mg kg^−1^) five times over the course of the 10 days (Fig. 1A). Matching previous reports, CpG-treated mice transiently lost weight following the administration of CpG (Fig. 1B). Treatment with CpG induced significant splenomegaly (Fig. 1C,D). Further, CpG administration resulted in a significant increase in plasma ferritin levels (Fig. 1E), a marker of inflammatory disease (Kell and Pretorius, 2014), and a significant increase in plasma cytokines, emulating cytokine storm associated with hyperinflammatory disease. CpG-treated mice had significantly elevated levels of plasma IFNγ, IL-18, IL-6, IL-10 and TNF compared to phosphate-buffered saline (PBS) injected controls (Fig. 1F-J), but plasma levels of IL-1β and IL-1α were below the limit of detection (data not shown). The CpG-induced mouse model of MAS has been well established as an appropriate model for ‘subclinical’ MAS, as it recapitulates a number of the pathologies associated with the disease and is worsened by removal of IL-18BP, i.e. the endogenous regulator of IL-18 signalling (Girard-Guyonvarc'h et al., 2018). However, in this mouse model, the source of IL-18 has not been reported. Therefore, we assessed if expression of components of the inflammasome is increased following CpG-induced hyperinflammation. Homogenised spleens from mice that had been repeatedly injected with CpG showed an enhanced expression of inflammasome components, such as NLRP3, caspase-1, GSDMD, pro-IL-1β and pro-IL-18, as well as caspase-1 activation and GSDMD-cleavage (Fig. 1K). These data showed that repeated administration of CpG produces markers of hyperinflammation and suggests that inflammasome signalling is upregulated during the development of MAS. This provides the first evidence that CpG-induced MAS leads to upregulation of inflammasome components, indicating a possible role for inflammasomes in disease pathogenesis.

We also wanted to assess the temporal dynamics of inflammasome upregulation to further dissect the pathogenesis of disease and ensure that 10 days of repeated CpG injections is optimal for analysis of inflammasome involvement in MAS. To examine this, we isolated tissue from mice at different timepoints in the development of CpG-induced MAS. Mice were injected with a single dose of CpG at day 0 and tissue was collected after 6h and 24h. Additionally, mice received cumulative injections of CpG on day 2, 4, 7 and 9, with tissue collection 24 h after each injection (i.e. on day 3, 5, 8 and 10, respectively). (Fig. S1A). Splenomegaly developed proportionally to the number of received CpG injections (Fig. S1B). CpG caused an initial hyperferritinaemic response within 24 h of administration, which remained elevated after two, three and four doses, until increasing further following the 5th dose (Fig. S1C). We observed different induction kinetics between cytokines. Plasma IFNγ, IL-6 and TNF peaked 24 h after CpG administration before decreasing over time, although the concentration of IFNγ remained elevated at all time points compared to non-treated animals (Fig. S1D,E,G). By contrast, plasma IL-18 levels exhibited initial acute elevation that dropped back to base level before another large and sustained increase at later stages of MAS development in response to the third and following injections (Fig. S1H); a similar response was seen for IL-10 (Fig. S1F). These data indicate that the model of repeated CpG injection presents with an initial acute inflammatory response, followed by the development of a more consistent hyperinflammatory phenotype, which recapitulates what is observed clinically with hyperinflammatory diseases, i.e. that inflammasome activation occurs later in the disease time course. Both liver and spleen homogenates indicated a dose-dependent increase in NLRP3, pro-caspase-1 and pro-IL-18 expression (Fig. S1I,J). The increase in inflammasome components in organ homogenates could be due to myeloid cell infiltration into the tissue. Histological analysis of the liver revealed that CpG-treated mice had a marked reduction in iron (Fe^3+^) in the liver over time, correlating with CpG doses (Fig. S1K). Perls Prussian Blue staining of spleen sections correlated with ferritin concentration in the plasma, indicative of Fe3^+^ sequestering and inflammation. These data suggest that upregulation of the NLRP3 inflammasome occurred later in the pathogenesis of CpG-induced MAS and that expression of inflammasome coincided with plasma IL-18 activity.

### The NLRP3 inflammasome is dispensable for the development of CpG-induced MAS

**Fig. 2.**
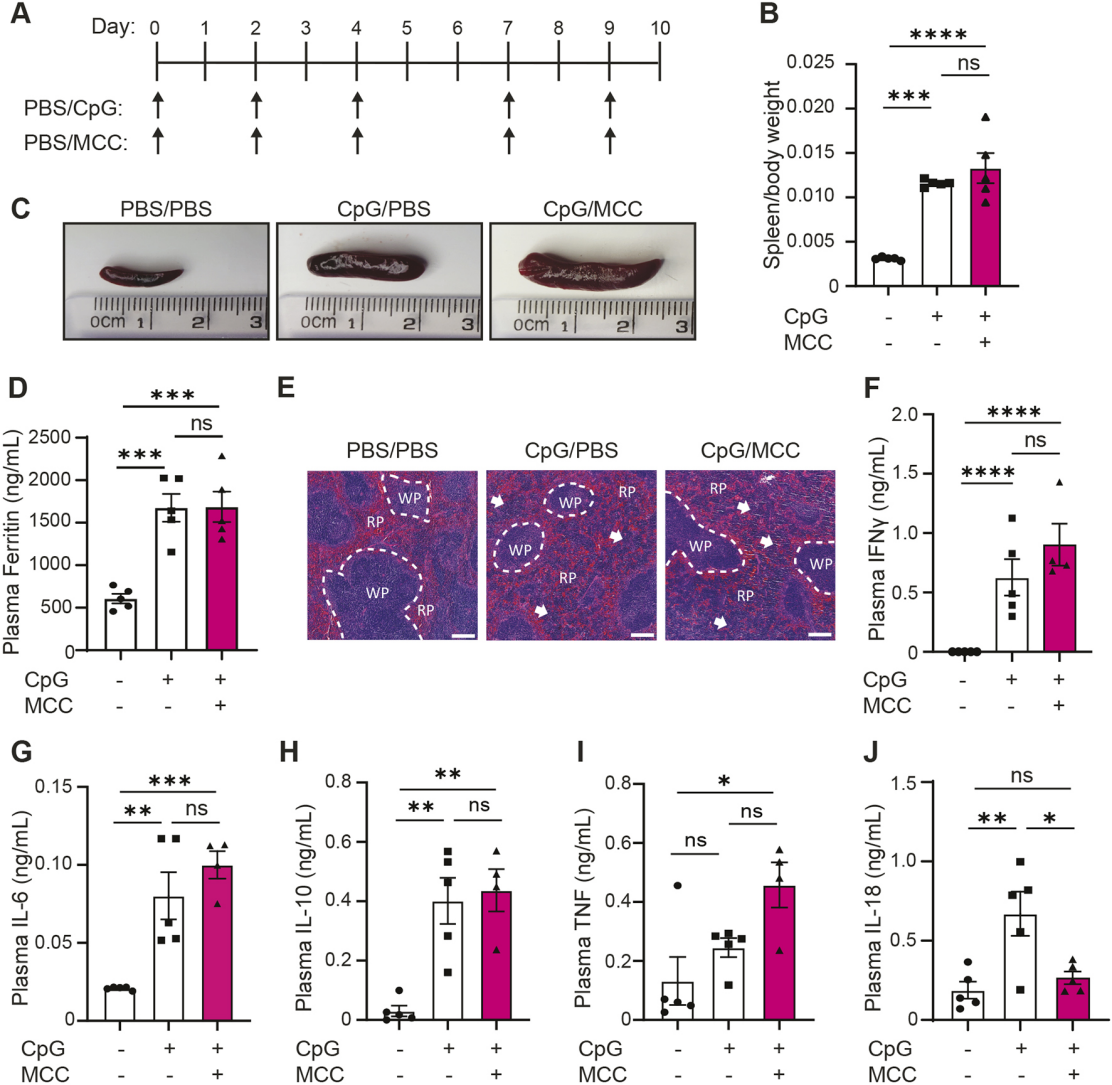
**The NLRP3 inflammasome is dispensable for development of CpG-induced hyperinflammation.** (A) Mice received five doses of either CpG (2 mg kg^−1^) or vehicle (PBS) on days 0, 2, 4, 7 and 9, as well as five doses of either MCC950 (MCC, 50 mg kg^−1^) or PBS on days 0, 2, 4, 7 and 9. (B) Ratio of spleen to body weight of mice injected with PBS (–/−), CpG and PBS (+/–) or CpG and MCC together (+/+) (*n*=5). (C) Representative images of spleens obtained from mice as described in A. (E) H&E staining of spleen in mice treated with PBS/PBS, CpG/PBS or CpG/MCC. RP, red pulp; WP, white pulp. Arrows denote changes to normal splenic architecture and perturbations to red pulp. Scale bars: 200 μm. (D,F-J) Plasma concentrations of ferritin (D), IFNγ (F), IL-6 (G), IL-10 (H) and TNF (I), IL-18 (J) in mice injected as defined in A (*n*=5). Data represent the mean±s.e.m. *P<0.05, **P<0.01, ***P<0.001, ****P<0.0001 (one-way ANOVA with Tukey's multiple comparisons test). ns, not significant.

To better understand the role of inflammasomes in CpG-induced MAS – and since we had observed an upregulation of the NLRP3 inflammasome following repeated injection of CpG – we then tested if the NLRP3 inflammasome is crucial for the development of CpG-induced hyperinflammation. To do this, mice were injected intraperitoneally (i.p.) with the NLRP3-specific inhibitor MCC950 (50 mg kg^−1^) (Coll et al., 2015) in tandem with CpG or PBS every 2 days over a 10-day period (Fig. 2A). As before, CpG treatment induced significant splenomegaly but this was not reduced following co-treatment with MCC950 (Fig. 2B,C). Further, CpG-induced hyperferritinaemia was not affected by concomitant treatment with MCC950 (Fig. 2D). Examination of splenic architecture revealed a disruption of normal red and white pulp morphology in animals that had been treated with CpG, which persisted when animals were treated with MCC950 (Fig. 2E). We then assessed if inflammasomes contribute to the development of the cytokine storm in CpG-induced hyperinflammation by analysing plasma cytokines in mice that had been treated with CpG and MCC950 (Fig. 2F-J). MCC950 treatment significantly reduced CpG-induced plasma IL-18 to similar levels as seen in PBS-injected animals (Fig. 2J), suggesting that the NLRP3 inflammasome is responsible for elevated levels of plasma IL-18. However, MCC950 treatment did not significantly alter production of IFNγ, IL-6, IL-10 or TNF in response to repeated administration of CpG (Fig. 2F-I), although we observed a trend towards an increase in plasma IFNγ (*P*=0.2810) and TNF (*P*=0.1113) concentrations (Fig. 2F). In addition, whilst we could still observe caspase-1 cleavage in liver homogenates following treatment with CpG, this was not significantly reduced in animals treated with MCC950 (Fig. S2A,B). Since MCC950 was effective at reducing plasma IL-18 levels, this suggests that the levels of MCC950 were insufficient to prevent inflammasome activation in tissues or that caspase-1 was alternatively processed. These results demonstrate that inhibition of the NLRP3 inflammasome was insufficient to prevent onset of CpG-induced hyperinflammatory disease.

### Inflammasome activation is not required to drive CpG-induced MAS

**Fig. 3.**
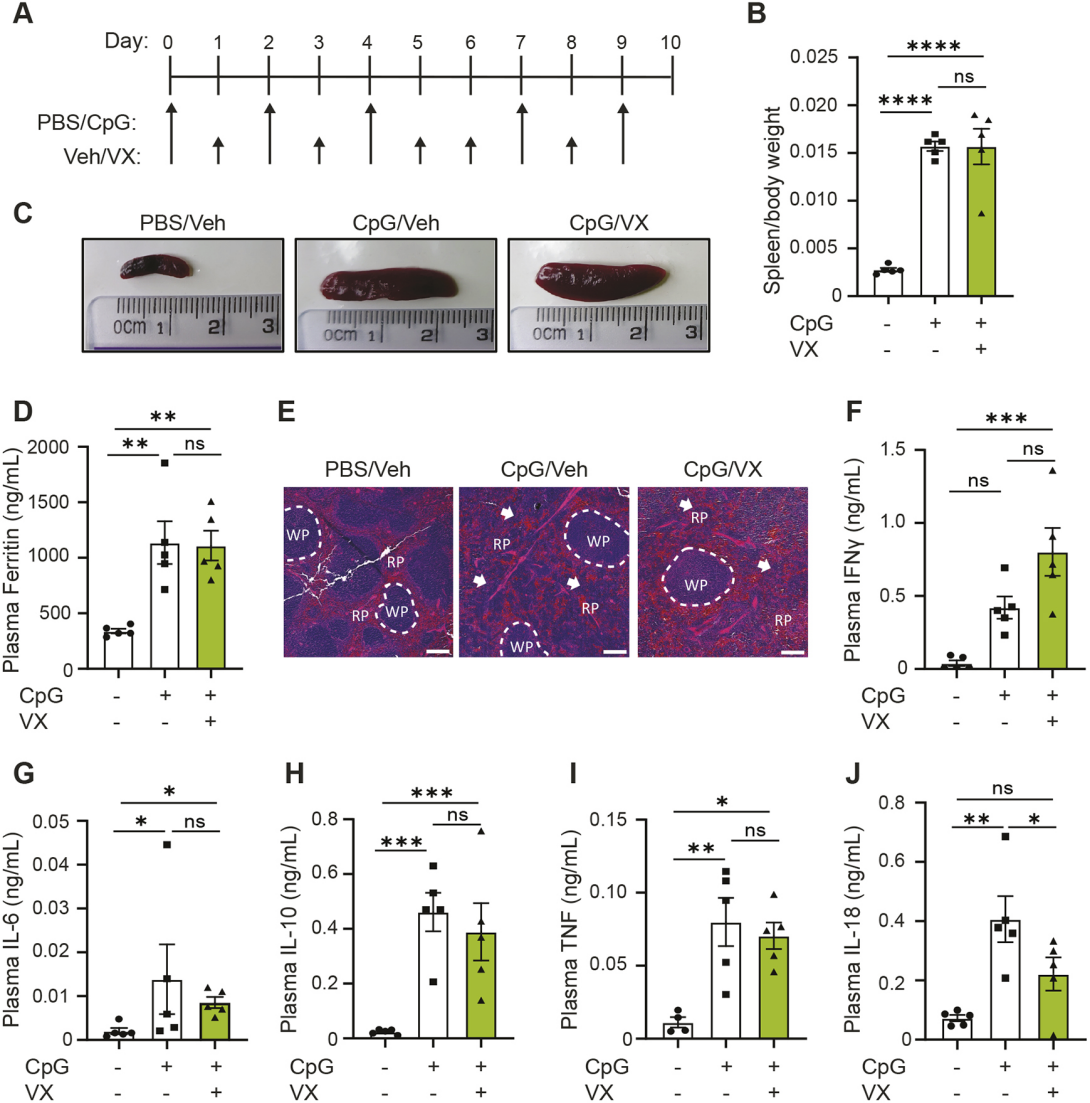
**Canonical inflammasome activation is dispensable for development of CpG-induced MAS.** (A) Mice received five doses of either CpG (2 mg kg^−1^) or vehicle (PBS) on days 0, 2, 4, 7 and 9, as well as daily injections (days 0-9) of either vehicle [Veh; 5% DMSO in PBS (v/v)] or VX-765 (VX, 100 mg kg^−1^). (B) Splenic weight normalised to body weight of mice injected with PBS and Veh (–/–), CpG and Veh (+/–) or CpG and VX (+/+) (*n*=5). (C) Representative images of spleens obtained from mice as described in A. (E) H&E staining of spleen from mice injected as described for A. RP, red pulp; WP, white pulp. Arrows denote changes to normal splenic architecture and perturbations to red pulp. Scale bars: 200 μm. (D,F-J) Plasma concentrations of ferritin (D), IFNγ (F), IL-6 (G), IL-10 (H), TNF (I) and IL-18 (J) in mice injected as described for A (*n*=5). Data represent the mean±s.e.m. *P<0.05, **P<0.01, ***P<0.001 (one-way ANOVA with Tukey's multiple comparisons test). ns, not significant.

Since inhibition of the NLRP3 inflammasome did not prevent hyperinflammatory disease, we then questioned if alternative inflammasomes can contribute towards the hyperinflammatory state. To test this, we used the caspase-1 inhibitor VX-765 (also known as Belnacasan). As mentioned previously, inflammasome activation leads to recruitment and cleavage of caspase-1 (Broz and Dixit, 2016; Martinon et al., 2002; Schroder and Tschopp, 2010), meaning that inhibition of caspase-1 activity results in pan-inflammasome inhibition (such as NLRP3, AIM2, NLRC4, NLRP1). To decipher the impact of inflammasome activation on MAS pathogenesis, we repeated our 10-day model of CpG-induced hyperinflammation with mice treated daily with the caspase-1 inhibitor VX-765 (Wannamaker et al., 2007) (100 mg kg^−1^, i.p.) (Fig. 3A). Caspase-1 inhibition with VX-765 did not affect development of splenomegaly (Fig. 3B,C), hyperferritinaemia (Fig. 3D) or prevent perturbations in splenic architecture (Fig. 3E), similar to what was observed after treatment with MCC950. Likewise, there were no significant changes to plasma levels of IFNγ, IL-6, IL-10 or TNF (Fig. 3F-I), but we observed a significant reduction in IL-18 (Fig. 3J) (*P*=0.0364) together with a non-significant trend to increased plasma IFNγ concentrations (*P*=0.0596) (Fig. 3F). Similar to treatment with MCC950, CpG-induced caspase-1 cleavage in the liver was not prevented by treatment with VX-765 (Fig. S2C,D). These data indicate that, apart plasma IL-18, inflammasomes are not essential in CpG-induced splenomegaly, elevated plasma ferritin, splenic tissue disruption or cytokine storm. Further, since caspase-1 inhibition exhibited the same phenotype as NLRP3 inhibition, this suggests that only the NLRP3 inflammasome is responsible for enhanced levels of plasma IL-18 in CpG-induced MAS.

### Inhibition of IL-1 receptor signalling with IL-1Ra does not impact the key parameters of MAS

**Fig. 4.**
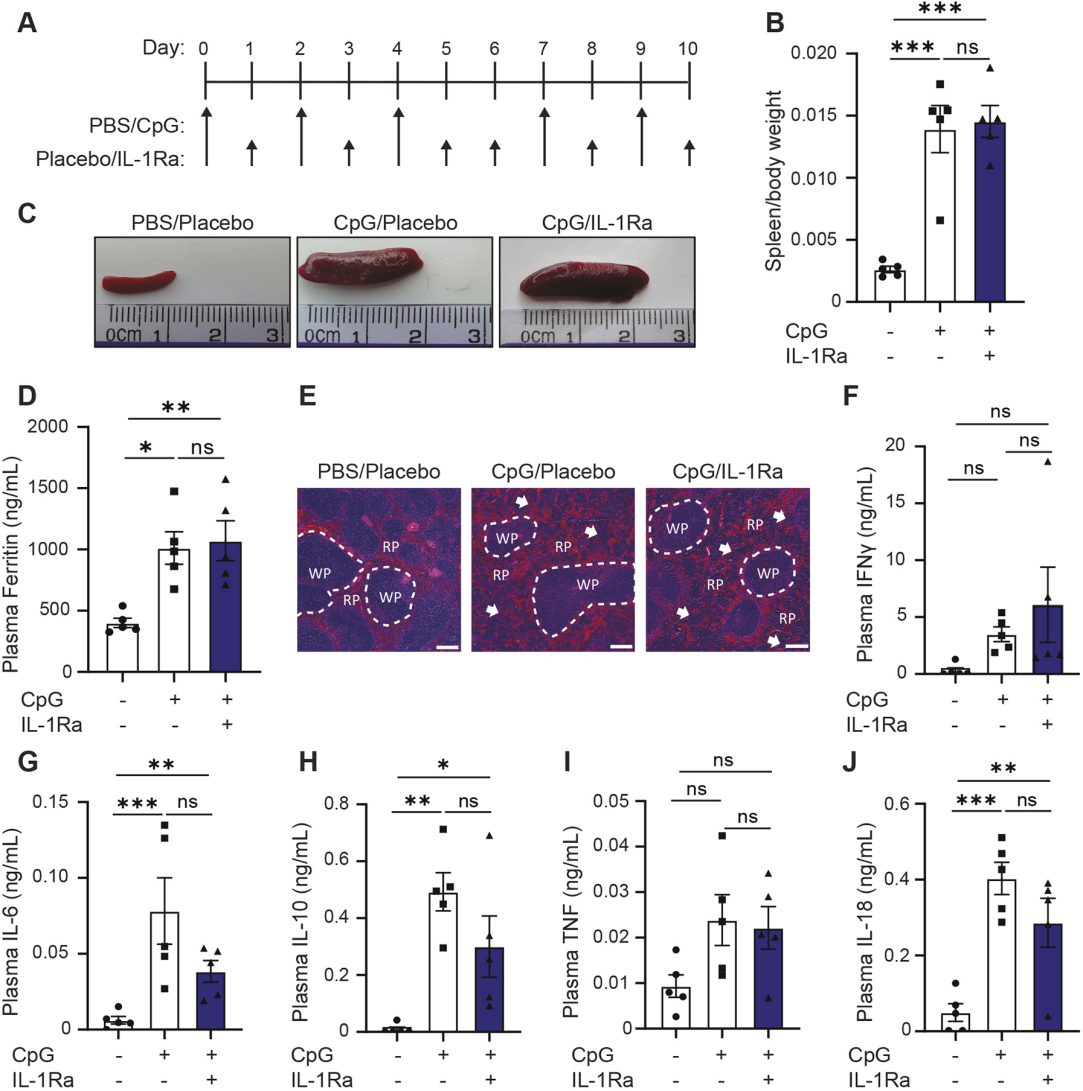
**IL-1Ra is not sufficient to prevent onset of hyperinflammation in CpG-treated mice.** (A) Mice received five doses of CpG (2 mg kg^−1^) or vehicle (PBS) on days 0, 2, 4, 7 and 9, as well as twice daily injections (days 0-10, at 9:00 and 18:00) of either placebo (see Materials and Methods) or anakinra (IL1Ra, 100 mg kg^−1^, twice daily). (B) Splenic weight normalised to body weight in mice treated with PBS and placebo (–/–), CpG and placebo (+/–) or CpG and IL-1Ra (+/+) (*n*=5). (C) Representative images of spleens obtained from mice as described in A. (E) H&E staining of spleen from mice injected as described for B. RP, red pulp; WP, white pulp. Arrows denote changes to normal splenic architecture and perturbations to red pulp. Scale bars: 200 μm. (D,F-J) Plasma concentrations of ferritin (D), IFNγ (F), IL-6 (G), IL-10 (H), IL-18 (I), and TNF (J) in mice injected as described for A (*n*=5). *P<0.05, **P<0.01, ***P<0.001 (one-way ANOVA with Tukey's multiple comparisons test). ns, not significant.

Following on from inflammasome inhibition studies, we sought to test the role of IL-1α/β signalling in this model and its function in the pathogenesis of MAS. Presently, inhibition of IL-1α/β signalling with recombinant IL-1Ra (anakinra), together with corticosteroids, has proven useful in the clinical treatment of MAS (Miettunen et al., 2011; Bruck et al., 2011; Ajeganova et al., 2020; Phadke et al., 2021; Naymagon, 2022; reviewed by Mehta et al., 2020a; Gleeson et al., 2022). As silencing of IL-1 signalling has shown some promise clinically, we tested anakinra in animals at a dose of 100 mg kg^−1^, injected subcutaneously twice daily. Following treatment of anakinra concomitant with induction of CpG-induced hyperinflammation, we examined the main parameters of disease to ascertain the role of IL-1α/β signalling in CpG-induced MAS (Fig. 4A). First, use of IL-1Ra was insufficient to prevent splenomegaly (Fig. 4B,C), indicating that IL-1 signalling was not a key driver of spleen enlargement. Second, we assessed the ability of anakinra to reduce inflammation in this model by examining plasma ferritin levels. Again, we observed that IL-1α/β did not drive pathogenesis of MAS, with IL-1Ra-treated animals displaying no difference in plasma ferritin levels compared to placebo-treated animals (Fig. 4D). Finally, IL-1Ra was unable to prevent CpG-associated splenic architecture disruptions (Fig. 4E). When examining plasma cytokine effects, there were no significant differences between CpG-treated mice and those who also received IL-1Ra (Fig. 4F-J) but, there was a trend towards a decrease in IL-6, IL-10 and IL-18 (Fig. 4G,H,J), indicative of general anti-inflammatory effects expected with IL-1Ra use. Plasma levels of IL-1α/β were below limit of detection (data not shown). Although IL-1Ra treatment is an efficacious treatment for MAS patients, it is not sufficient to prevent CpG-induced hyperinflammation in these animals.

## DISCUSSION

Inflammasome-derived cytokines have been proposed to be involved in hyperinflammation and CSS (Mazodier et al., 2005; Yasin et al., 2020a; Weiss et al., 2018; Shimizu et al., 2010; Krei et al., 2021; Crayne et al., 2019; Phadke et al., 2021; Mehta et al., 2020a; Ajeganova et al., 2020; Vora et al., 2021) but the role of inflammasomes in the progression of hyperinflammation is not fully understood. Therefore, we sought to identify the role of inflammasomes in the induction of hyperinflammation by using a mouse model of CpG-induced MAS. Here, we show that components of the NLRP3 inflammasome were upregulated following induction of CpG-induced hyperinflammation, and that inflammasomes were critical for the induction of IL-18, a key cytokine involved in the clinical manifestations of MAS (Shiga et al., 2021; Krei et al., 2021; Weiss et al., 2018). However, blockade of NLRP3, caspase-1 or IL-1 signalling was not sufficient to prevent the development of several features of hyperinflammatory disease, such as hyperferritinaemia and splenomegaly, suggesting that these traits occur independent of the NLRP3 inflammasome.

This is the first time inflammasomes have been studied during the development of CpG-induced MAS. However, inflammasomes have already been directly implicated in NLRC4-MAS (Canna et al., 2014, 2017; Barsalou et al., 2018; Chear et al., 2020). Gain-of-function mutations in the NLRC4 inflammasome, leading to aberrant activation of the NLRC4 inflammasome have been characterised as ‘IL-18opathies’ as there is such a strong IL-18 signature associated with these mutations (Canna et al., 2014, 2017; Chear et al., 2020). This pathology is very similar to that observed in other instances of hyperinflammation, including MAS not caused by mutations in NLRC4. In this study, we demonstrated that in CpG-induced MAS, inhibition of the NLRP3 inflammasome resulted in a reduction in plasma levels of IL-18, indicating that the NLRP3 inflammasome was the source of raised amounts of IL-18 in CpG-induced MAS. Thus, it is possible that the NLRP3 inflammasome is a source of IL-18 in clinical instances of MAS without NLRC4 mutations. Further evidence for inflammasome activation in patients with MAS is evident as inhibiting IL-1 signalling has proven efficacious in the management of MAS flares in SJIA and AOSD. High-dose IL-1Ra (anakinra) treatment has proven to be effective in patients with refractory MAS (Kahn and Cron, 2013; Mehta et al., 2020a,b; Aytac et al., 2016), as well as beneficial in the treatment of MAS in patients diagnosed with sepsis (Shakoory et al., 2016). However, our data did not support the use of IL-1Ra in the context of this mouse model of hyperinflammation, potentially because these animals had no underlying chronic inflammatory disease, infection or existing comorbidities.

In patients, IL-18 and IFNγ signalling is understood to be essential for driving disease pathogenesis, whereas the importance of IL-18 and IFNγ in animal models of MAS is less clear. We show that inhibition of the NLRP3 inflammasome in CpG-induced MAS resulted in a significant reduction in plasma IL-18 levels but did not alter several features of MAS pathogenesis. This supports previous studies that have characterised the effect inhibition of IL-18 receptor (IL-18R) has on CpG-induced MAS severity (Girard-Guyonvarc'h et al., 2018). Our data demonstrated that IL-18 is not essential for the development of characteristics associated with hyperinflammatory disease, including hyperferritinaemia, splenomegaly and cytokine storm, mirroring the results observed in response to direct inhibition of IL-18 signalling (Girard-Guyonvarc'h et al., 2018). Although IL-18 is thought to be a key cytokine involved in the clinical pathogenesis of MAS, it is not essential in this model. Further, our data demonstrate that in CpG-induced MAS, the amount of IL-18 and IFNγ appeared to be uncoupled, such as inhibition of the NLRP3 inflammasome was sufficient to reduce plasma levels of IL-18, whilst levels of plasma IFNγ remained elevated. Originally, IL-18 was coined as IFNγ-inducing factor (IGIF) (Tone et al., 1997; Gu et al., 1997) and, although its role in immunity has become more widely studied (Dinarello et al., 2013; Ihim et al., 2022), induction of IFNγ remains a key role for IL-18. Our data indicated that IL-18 is not an essential driver of IFNγ in CpG-induced MAS. Similarly, blockade of IL-18 does not significantly impact plasma concentration of IFNγ in CpG-induced MAS (Girard-Guyonvarc'h et al., 2018). Together, these findings highlight that, during cytokine storm, other mechanisms may be responsible for induction of IFNγ. These could include cytokines, such as type I IFN, IL-15, IL-2 and IL-12, all of which have been demonstrated to drive IFNγ production *in vitro* and *in vivo* (Cui et al., 2024; Kwon et al., 2019), with elevated IL-12 levels already observed in cytokine storm triggered by CpG-induced MAS (Behrens et al., 2011; Gao et al., 2021). Interestingly, inhibition of IFNγ has been shown to be effective in reducing several of the pathologies associated with hyperinflammation in CpG-induced MAS, including splenomegaly, anaemia, thrombocytopenia and hypercytokinaemia (Behrens et al., 2011; Gao et al., 2021). Furthermore, inhibition of IFNγ in CpG-induced MAS results in reduction of MAS severity in IL-18BP-knockout mice (Girard-Guyonvarc'h et al., 2018), indicating that IFNγ is an important player in disease pathogenesis. Although not significant, our data suggest a trend towards an increase in plasma levels of IFNγ in animals with reduced plasma levels of IL-18, alluding to a change in the signature of the cytokine storm.

Although our data suggest that inflammasomes and IL-18 have a non-essential role in several manifestations of MAS, we must acknowledge that our study was performed using young and healthy mice without any pre-existing comorbidities. In humans, MAS flares commonly occur with an underlying inflammatory disease, such as SJIA or AOSD (Ravelli et al., 2016; Iwamoto, 2007; Crayne et al., 2019; Lenert and Yao, 2016), which exhibit a significant level of basal inflammation that might be crucial to fully recapitulate the disease. Clinically, IL-18 has been demonstrated to be a cytokine crucial in MAS pathogenesis as: 1) many patients presenting with hyperinflammatory disease present with significantly elevated plasma levels of IL-18 (Yasin et al., 2020a; Kawashima et al., 2001) and, 2) plasma concentration of IL-18 correlates strongly with disease severity (Weiss et al., 2018) and, 3) targeting IL-18 signalling seems to be a promising strategy to treat MAS (Yasin et al., 2020b). By contrast, our data suggest that IL-18 is not important in CpG-induced MAS in young and healthy animals, since inhibition of NLRP3 or caspase-1 was able to significantly reduce plasma IL-18 levels without preventing the development of MAS. Therefore, we propose that – although the NLRP3 inflammasome was necessary to produce plasma IL-18 in this model of MAS – additional intrinsic mechanisms regulate IL-18-dependent inflammation in healthy young mice, suggesting that our results do not necessarily reflect those observed in patients with hyperinflammatory disease. Similarly, inhibition of the IL-18 receptor is insufficient to alter CpG-induced MAS in young wild-type mice but does prevent the development of a more-severe MAS phenotype observed in IL-18BP-knockout mice (Girard-Guyonvarc'h et al., 2018), indicating that endogenous regulation of IL-18 is paramount for controlling disease severity (Weiss et al., 2018). Importantly, since we have identified the NLRP3 inflammasome as the source of IL-18 in CpG-induced MAS, targeting the NLRP3 inflammasome in patients with MAS – where IL-18 signalling is crucial – could be an effective treatment to alleviate the disease. To gain further insight into involvement of the inflammasome in a more-severe disease phenotype, mirroring what is observed in patients with MAS flares, future studies using older animals, or those with pre-existing conditions, such as obesity, chronic inflammatory disease or infection, are required.

In summary, we identified that the NLRP3 inflammasome is activated in CpG-induced hyperinflammation and critical for enhanced production of IL-18, a key driver of hyperinflammatory disease in patients diagnosed with MAS. However, in the CpG-induced model of MAS, NLRP3 and IL-18 were not required for the characteristic manifestations of hyperinflammation, including splenomegaly and hyperferritinaemia. Our study sheds new light on the dynamics of hyperinflammation observed in MAS, suggesting that, although the NLRP3 inflammasome is essential for IL-18 production, it is not a driving factor in the pathogenesis of CpG-induced MAS. Since IL-18 is established to be crucial in the clinical development of MAS, the NLRP3 inflammasome could be an effective target to treat hyperinflammation and certainly validates further research.

## MATERIALS AND METHODS

### Animals

Male 8–12-week-old C57BL/6J mice (Charles River Laboratories, UK) were used in all experiments. Animals were housed in individually ventilated cages, with temperature and humidity maintained at 20–24°C and 45−65%, respectively. Animals were housed in a room with a 12 h light–dark cycle. All animal experiments were carried out under the authority of a UK Home Office Project Licence and reported according to the ARRIVE guidelines for experiments involving animals (McGrath and Lilley, 2015).

### Induction of MAS by CpG

Mice were treated 5× over the course of 10 days with 2 mg kg^−1^ of either CpG oligonucleotide 1826 (ODN 1826, hereafter referred to as CpG) [synthesised by Integrated DNA technologies (IDT), sequence 5′-T*C*C*A*T*G*A*C*G*T*T*C*C*T*G*A*C*G*T*T-3′, where * indicates phosphorothioate modification] or with vehicle control [sterile phosphate-buffered saline (PBS), 10 μl g^−1^], as described previously (Behrens et al., 2011). Mice received CpG or PBS by intraperitoneal (i.p) injection on days 0, 2, 4, 7 and 9 of the protocol. Animals were weighed daily and culled on day 10 (24 hours post injection) or at the indicated time-point for time course experiments. The following treatments were given as i.p. injections unless stated otherwise: NLRP3 inhibitor MCC950 (50 mg kg^−1^ in PBS; #PZ0280, Sigma Aldrich), (for details see Coll et al., 2015) at the same time as CpG; vehicle controls received 10 μl/g PBS. The caspase-1 inhibitor VX-765 (also known as Belnacasan; #7143/10, Tocris Bioscience) was injected daily at 100 mg kg^−1^ in 5% (v/v) DMSO in PBS (for details see Wannamaker et al., 2007); vehicle controls received 10 μl/g 5% (v/v) DMSO in PBS. Recombinant IL-1Ra (also known as anakinra) was administered twice daily by sub-cutaneous injection at 100 mg kg^−1^ in placebo (638 mM polysorbate 80, 5.2 mM sodium citrate, 112 mM sodium chloride, 45.4 μM disodium EDTA pH 6.5, dH_2_O; Sobi, Sweden) (for details see Sun et al., 2017). A high dose of IL-1Ra via subcutaneous injection has been recommended for clinical treatment of CSS (Kahn and Cron, 2013; Carter et al., 2019). Vehicle controls received 10 μl/g placebo. For the duration of the experiment, researchers were unaware of the experimental treatment.

### Tissue collection

On day 10, mice were deeply anaesthetised with 2.5% isoflurane (Isofane, VM no.: 37071/4001, Henry Schein) under 33% O_2_ and 67% NO_2_, and blood was taken via cardiac puncture. Blood was centrifuged at 1500***g*** for 15 min, plasma removed and centrifuged again at 14,000***g*** for 3 min. Plasma was then aliquoted and stored at −80°C for analysis. Following cardiac puncture, mice were perfused with PBS, their spleen removed and weight recorded, and divided for further analysis. Liver was removed and dissected for further analysis. A proportion of livers and spleens were snap frozen in dry ice for western blot analysis, and another portion was drop fixed in 4% paraformaldehyde (PFA) and then embedded in paraffin.

### ELISA

Sandwich ELISA was used to establish plasma concentrations of IL-18 (#BMS618-3, Invitrogen, Thermo Fisher Scientific) and ferritin (#ab157713, Abcam), carried out and read at 450-570 nm according to the manufacturer's instructions.

### Multiplex cytokine analysis assay

The concentration of IFNγ, TNF, IL-10, IL-6, IL-1α and IL-1β was measured by using the LEGENDplex flow-based 13-plex mouse inflammation panel kit from Biolegend (#740446) according to the manufacturer's instructions. LEGENDplex results were obtained by using BD FACSVerse (BD Biosciences).

### Tissue homogenisation

Livers and spleens were snap frozen on dry ice immediately following isolation, and stored at −80°C prior to homogenisation. Spleens were homogenised in RIPA lysis buffer [150 mM sodium chloride, 1.0% (v/v) NP-40, 0.5% (w/v) sodium deoxycholate, 0.1% (w/v) sodium dodecyl sulfate, 50 mM Tris pH 8.0, dH_2_O] containing protease inhibitor cocktail (#539131-10, Sigma). Livers were homogenised in NP-40 lysis buffer [0.5% (v/v) NP-40, 150 mM sodium chloride, 2 mM EDTA, 50 mM Tris pH 8.0, dH_2_O] containing protease inhibitor cocktail (#539131-10, Sigma). Protein concentration of homogenates was determined using BCA (bicinchoninic) protein assay kit (#23225, Thermo Fisher Scientific). BCA assay was used to the determine protein concentration of each sample, samples were used at a concentration of 175μg for western blotting.

### Western blotting

Laemmli buffer (5×) was added to samples that were then boiled at 95°C for 10 min before being analysed using by SDS-PAGE. Following protein separation, gels were transferred onto nitrocellulose or PVDF membranes using the Trans-Blot® Turbo Transfer™ System (Bio-Rad). Membranes were blocked in 5% (w/v) milk in PBS 0.1% (v/v) Tween-20 (PBST) at room temperature for 1 h, washed with PBST and incubated overnight with antibodies. The following antibodies were used: rabbit-anti-mouse IL-18 (1:1000 dilution; #E9P50, #57058, Cell Signalling Technology), rabbit-anti-mouse caspase-1 p10 (1:1000 dilution; #EPR16883, #ab179515 Abcam), mouse-anti-mouse NLRP3 (1:1000 dilution; Cryo2, #AG-20B-0014, Adipogen), rabbit-anti-mouse GSDMD (1:1000 dilution; #EPR19828, #ab209845, Abcam), or goat-anti-mouse IL-1β (1:800 dilution; #AF-401-NA, R&D Systems), primary antibodies were diluted in 5% (w/v) BSA in PBST. The membranes were washed and incubated at room temperature for 1 h with secondary antibodies rabbit anti-mouse IgG (#P026002-2, Agilent) or goat anti-rabbit IgG (#P044801-2, Agilent) diluted 1:1000 in 5% (w/v) BSA in PBST. Proteins were then visualised by using the Amersham ECL Prime Western Blotting Detection Reagent (Cytiva #RPN2236), and G:BOX (Syngene) and Genesys software. β-Actin was used as a loading control [antibody: 1:20,000 dilution 5% (w/v) BSA in PBST; #A3854, Sigma-Aldrich].

### Histology

Spleen sections (5 µm) cut by using a paraffin rotary microtome (Leica), were stained with Haematoxylin and Eosin Y (H&E; Thermo Fisher Scientic) Coverslips were applied using DPX mountant (#06522, Sigma-Aldrich). Spleen sections were stained for Fe3^+^ with Perls Prussian Blue Stain Kit (#65692, Abcam) according to the manufacturer's instructions. Sections were scanned on a SlideScanner (Panoramic P250) and analysed using CaseViewer. CaseViewer software (both 3Dhistech Ltd.).

### Statistical analysis

Data were analysed using GraphPad PRISM 9 software (GraphPad Software Inc. CA, USA). Results are presented as the mean±s.e.m. Equal variance and normality were assessed by using the Shapiro–Wilk test. To compare two data sets (PBS versus CpG), unpaired Student's *t*-test was chosen. For data with two factors (PBS/CpG ±vehicle/treatment), one-way ANOVA with Tukey's multiple comparison test was performed. Non-parametric data were transformed before statistical analysis. Accepted levels of significance were *P<0.05, **P<0.01, ***P<0.001 and ****P<0.0001. Studies were conducted on groups of three or five animals, where *n* represents an individual animal.

## Supplementary Material

10.1242/dmm.050762_sup1 Supplementary information

## References

[DMM050762C1] Abdelkefi, A., Jamil, W. B., Torjman, L., Ladeb, S., Ksouri, H., Lakhal, A., Hassen, A. B., Abdeladhim, A. B. and Othman, T. B. (2009). Hemophagocytic syndrome after hematopoietic stem cell transplantation: a prospective observational study. *Int. J. Hematol.* 89, 368-373. 10.1007/s12185-009-0261-1 19252966

[DMM050762C2] Ajeganova, S., De Becker, A. and Schots, R. (2020). Efficacy of high-dose anakinra in refractory macrophage activation syndrome in adult-onset Still's disease: when dosage matters in overcoming secondary therapy resistance. *Ther. Adv. Musculoskelet Dis.* 12, 1759720X20974858. 10.1177/1759720X20974858 PMC7692351 33281955

[DMM050762C3] Aytac, S., Batu, E. D., Unal, S., Bilginer, Y., Cetin, M., Tuncer, M., Gumruk, F. and Ozen, S. (2016). Macrophage activation syndrome in children with systemic juvenile idiopathic arthritis and systemic lupus erythematosus. *Rheumatol. Int.* 36, 1421-1429. 10.1007/s00296-016-3545-9 27510530

[DMM050762C4] Barsalou, J., Blincoe, A., Fernandez, I., Dal-Soglio, D., Marchitto, L., Selleri, S., Haddad, E., Benyoucef, A. and Touzot, F. (2018). Rapamycin as an adjunctive therapy for NLRC4 associated macrophage activation syndrome. *Front. Immunol.* 9, 2162. 10.3389/fimmu.2018.02162 30319625 PMC6166634

[DMM050762C5] Behrens, E. M., Canna, S. W., Slade, K., Rao, S., Kreiger, P. A., Paessler, M., Kambayashi, T. and Koretzky, G. A. (2011). Repeated TLR9 stimulation results in macrophage activation syndrome-like disease in mice. *J. Clin. Invest.* 121, 2264-2277. 10.1172/JCI43157 21576823 PMC3104738

[DMM050762C6] Broz, P. and Dixit, V. M. (2016). Inflammasomes: mechanism of assembly, regulation and signalling. *Nat. Rev. Immunol.* 16, 407-420. 10.1038/nri.2016.58 27291964

[DMM050762C7] Bruck, N., Suttorp, M., Kabus, M., Heubner, G., Gahr, M. and Pessler, F. (2011). Rapid and sustained remission of systemic juvenile idiopathic arthritis-associated macrophage activation syndrome through treatment with anakinra and corticosteroids. *J. Clin. Rheumatol.* 17, 23-27. 10.1097/RHU.0b013e318205092d 21169853

[DMM050762C8] Canna, S. W., Wrobel, J., Chu, N., Kreiger, P. A., Paessler, M. and Behrens, E. M. (2013). Interferon-gamma mediates anemia but is dispensable for fulminant toll-like receptor 9-induced macrophage activation syndrome and hemophagocytosis in mice. *Arthritis. Rheum.* 65, 1764-1775. 10.1002/art.37958 23553372 PMC3708494

[DMM050762C9] Canna, S. W., De Jesus, A. A., Gouni, S., Brooks, S. R., Marrero, B., Liu, Y., Dimattia, M. A., Zaal, K. J., Sanchez, G. A., Kim, H. et al. (2014). An activating NLRC4 inflammasome mutation causes autoinflammation with recurrent macrophage activation syndrome. *Nat. Genet.* 46, 1140-1146. 10.1038/ng.3089 25217959 PMC4177369

[DMM050762C10] Canna, S. W., Girard, C., Malle, L., De Jesus, A., Romberg, N., Kelsen, J., Surrey, L. F., Russo, P., Sleight, A., Schiffrin, E. et al. (2017). Life-threatening NLRC4-associated hyperinflammation successfully treated with IL-18 inhibition. *J. Allergy Clin. Immunol.* 139, 1698-1701. 10.1016/j.jaci.2016.10.022 27876626 PMC5846100

[DMM050762C11] Carter, S. J., Tattersall, R. S. and Ramanan, A. V. (2019). Macrophage activation syndrome in adults: recent advances in pathophysiology, diagnosis and treatment. *Rheumatology (Oxf.)* 58, 5-17. 10.1093/rheumatology/key006 29481673

[DMM050762C12] Chear, C. T., Nallusamy, R., Canna, S. W., Chan, K. C., Baharin, M. F., Hishamshah, M., Ghani, H., Ripen, A. M. and Mohamad, S. B. (2020). A novel de novo NLRC4 mutation reinforces the likely pathogenicity of specific LRR domain mutation. *Clin. Immunol.* 211, 108328. 10.1016/j.clim.2019.108328 31870725

[DMM050762C13] Coll, R. C., Robertson, A. A., Chae, J. J., Higgins, S. C., Munoz-Planillo, R., Inserra, M. C., Vetter, I., Dungan, L. S., Monks, B. G., Stutz, A. et al. (2015). A small-molecule inhibitor of the NLRP3 inflammasome for the treatment of inflammatory diseases. *Nat. Med.* 21, 248-255. 10.1038/nm.3806 25686105 PMC4392179

[DMM050762C14] Crayne, C. B., Albeituni, S., Nichols, K. E. and Cron, R. Q. (2019). The immunology of macrophage activation syndrome. *Front. Immunol.* 10, 119. 10.3389/fimmu.2019.00119 30774631 PMC6367262

[DMM050762C15] Cui, A., Huang, T., Li, S., Ma, A., Perez, J. L., Sander, C., Keskin, D. B., Wu, C. J., Fraenkel, E. and Hacohen, N. (2024). Dictionary of immune responses to cytokines at single-cell resolution. *Nature* 625, 377-384. 10.1038/s41586-023-06816-9 38057668 PMC10781646

[DMM050762C16] Dinarello, C. A. (1996). Biologic basis for interleukin-1 in disease. *Blood* 87, 2095-2147. 10.1182/blood.V87.6.2095.bloodjournal8762095 8630372

[DMM050762C17] Dinarello, C. A., Novick, D., Kim, S. and Kaplanski, G. (2013). Interleukin-18 and IL-18 binding protein. *Front. Immunol.* 4, 289. 10.3389/fimmu.2013.00289 24115947 PMC3792554

[DMM050762C18] Fajgenbaum, D. C. and June, C. H. (2020). Cytokine storm. *N. Engl. J. Med.* 383, 2255-2273. 10.1056/NEJMra2026131 33264547 PMC7727315

[DMM050762C19] Filipovich, A. H. and Chandrakasan, S. (2015). Pathogenesis of hemophagocytic lymphohistiocytosis. *Hematol. Oncol. Clin. North Am.* 29, 895-902. 10.1016/j.hoc.2015.06.007 26461149

[DMM050762C20] Gabay, C., Fautrel, B., Rech, J., Spertini, F., Feist, E., Kotter, I., Hachulla, E., Morel, J., Schaeverbeke, T., Hamidou, M. A. et al. (2018). Open-label, multicentre, dose-escalating phase II clinical trial on the safety and efficacy of tadekinig alfa (IL-18BP) in adult-onset Still's disease. *Ann. Rheum. Dis.* 77, 840-847. 10.1136/annrheumdis-2017-212608 29472362 PMC5965361

[DMM050762C21] Gao, D. K., Salomonis, N., Henderlight, M., Woods, C., Thakkar, K., Grom, A. A., Thornton, S., Jordan, M. B., Wikenheiser-Brokamp, K. A. and Schulert, G. S. (2021). IFN-gamma is essential for alveolar macrophage-driven pulmonary inflammation in macrophage activation syndrome. *JCI Insight* 6, e147593. 10.1172/jci.insight.147593 34314387 PMC8492332

[DMM050762C22] Girard-Guyonvarc'h, C., Palomo, J., Martin, P., Rodriguez, E., Troccaz, S., Palmer, G. and Gabay, C. (2018). Unopposed IL-18 signaling leads to severe TLR9-induced macrophage activation syndrome in mice. *Blood* 131, 1430-1441. 10.1182/blood-2017-06-789552 29295842

[DMM050762C23] Girard, C., Rech, J., Brown, M., Allali, D., Roux-Lombard, P., Spertini, F., Schiffrin, E. J., Schett, G., Manger, B., Bas, S. et al. (2016). Elevated serum levels of free interleukin-18 in adult-onset Still's disease. *Rheumatology (Oxf.)* 55, 2237-2247. 10.1093/rheumatology/kew300 27616144

[DMM050762C24] Gleeson, T. A., Nordling, E., Kaiser, C., Lawrence, C. B., Brough, D., Green, J. P. and Allan, S. M. (2022). Looking into the IL-1 of the storm: are inflammasomes the link between immunothrombosis and hyperinflammation in cytokine storm syndromes? *Discov. Immunol.* 1, kyac005. 10.1093/discim/kyac005 38566906 PMC10917224

[DMM050762C25] Gu, Y., Kuida, K., Tsutsui, H., Ku, G., Hsiao, K., Fleming, M. A., Hayashi, N., Higashino, K., Okamura, H., Nakanishi, K. et al. (1997). Activation of interferon-gamma inducing factor mediated by interleukin-1beta converting enzyme. *Science* 275, 206-209. 10.1126/science.275.5297.206 8999548

[DMM050762C26] Ihim, S. A., Abubakar, S. D., Zian, Z., Sasaki, T., Saffarioun, M., Maleknia, S. and Azizi, G. (2022). Interleukin-18 cytokine in immunity, inflammation, and autoimmunity: biological role in induction, regulation, and treatment. *Front. Immunol.* 13, 919973. 10.3389/fimmu.2022.919973 36032110 PMC9410767

[DMM050762C27] Iwamoto, M. (2007). Macrophage activation syndrome associated with adult-onset Still's disease. *Nihon Rinsho Meneki Gakkai Kaishi* 30, 428-431. 10.2177/jsci.30.428 18174671

[DMM050762C28] Jelusic, M., Lukic, I. K., Tambic-Bukovac, L., Dubravcic, K., Malcic, I., Rudan, I. and Batinic, D. (2007). Interleukin-18 as a mediator of systemic juvenile idiopathic arthritis. *Clin. Rheumatol.* 26, 1332-1334. 10.1007/s10067-006-0474-0 17597334

[DMM050762C29] Jordan, M. B., Hildeman, D., Kappler, J. and Marrack, P. (2004). An animal model of hemophagocytic lymphohistiocytosis (HLH): CD8+ T cells and interferon gamma are essential for the disorder. *Blood* 104, 735-743. 10.1182/blood-2003-10-3413 15069016

[DMM050762C30] Kahn, P. J. and Cron, R. Q. (2013). Higher-dose Anakinra is effective in a case of medically refractory macrophage activation syndrome. *J. Rheumatol.* 40, 743-744. 10.3899/jrheum.121098 23637382

[DMM050762C31] Kawashima, M., Yamamura, M., Taniai, M., Yamauchi, H., Tanimoto, T., Kurimoto, M., Miyawaki, S., Amano, T., Takeuchi, T. and Makino, H. (2001). Levels of interleukin-18 and its binding inhibitors in the blood circulation of patients with adult-onset Still's disease. *Arthritis. Rheum.* 44, 550-560. 10.1002/1529-0131(200103)44:3<550::AID-ANR103>3.0.CO;2-5 11263769

[DMM050762C32] Kayagaki, N., Kornfeld, O. S., Lee, B. L., Stowe, I. B., O'rourke, K., Li, Q., Sandoval, W., Yan, D., Kang, J., Xu, M. et al. (2021). NINJ1 mediates plasma membrane rupture during lytic cell death. *Nature* 591, 131-136. 10.1038/s41586-021-03218-7 33472215

[DMM050762C33] Kell, D. B. and Pretorius, E. (2014). Serum ferritin is an important inflammatory disease marker, as it is mainly a leakage product from damaged cells. *Metallomics* 6, 748-773. 10.1039/C3MT00347G 24549403

[DMM050762C34] Krei, J. M., Moller, H. J. and Larsen, J. B. (2021). The role of interleukin-18 in the diagnosis and monitoring of hemophagocytic lymphohistiocytosis/macrophage activation syndrome - a systematic review. *Clin. Exp. Immunol.* 203, 174-182. 10.1111/cei.13543 33128796 PMC7806447

[DMM050762C35] Kwon, K. W., Kim, S. J., Kim, H., Kim, W. S., Kang, S. M., Choi, E., Ha, S. J., Yoon, J. H. and Shin, S. J. (2019). IL-15 generates IFN-γ-producing cells reciprocally expressing lymphoid-myeloid markers during dendritic cell differentiation. *Int. J. Biol. Sci.* 15, 464-480. 10.7150/ijbs.25743 30745835 PMC6367559

[DMM050762C36] Latz, E., Xiao, T. S. and Stutz, A. (2013). Activation and regulation of the inflammasomes. *Nat. Rev. Immunol.* 13, 397-411. 10.1038/nri3452 23702978 PMC3807999

[DMM050762C37] Lenert, A. and Yao, Q. (2016). Macrophage activation syndrome complicating adult onset Still's disease: a single center case series and comparison with literature. *Semin. Arthritis Rheum.* 45, 711-716. 10.1016/j.semarthrit.2015.11.002 26672682

[DMM050762C38] Liu, A. C., Yang, Y., Li, M. T., Jia, Y., Chen, S., Ye, S., Zeng, X. Z., Wang, Z., Zhao, J. X., Liu, X. Y. et al. (2018). Macrophage activation syndrome in systemic lupus erythematosus: a multicenter, case-control study in China. *Clin. Rheumatol.* 37, 93-100. 10.1007/s10067-017-3625-6 28409239

[DMM050762C39] Martinon, F., Burns, K. and Tschopp, J. (2002). The inflammasome: a molecular platform triggering activation of inflammatory caspases and processing of proIL-beta. *Mol. Cell* 10, 417-426. 10.1016/S1097-2765(02)00599-3 12191486

[DMM050762C40] Mazodier, K., Marin, V., Novick, D., Farnarier, C., Robitail, S., Schleinitz, N., Veit, V., Paul, P., Rubinstein, M., Dinarello, C. A. et al. (2005). Severe imbalance of IL-18/IL-18BP in patients with secondary hemophagocytic syndrome. *Blood* 106, 3483-3489. 10.1182/blood-2005-05-1980 16020503 PMC1895045

[DMM050762C41] Mcgrath, J. C. and Lilley, E. (2015). Implementing guidelines on reporting research using animals (ARRIVE etc.): new requirements for publication in BJP. *Br. J. Pharmacol.* 172, 3189-3193. 10.1111/bph.12955 25964986 PMC4500358

[DMM050762C42] Mehta, P., Cron, R. Q., Hartwell, J., Manson, J. J. and Tattersall, R. S. (2020a). Silencing the cytokine storm: the use of intravenous anakinra in haemophagocytic lymphohistiocytosis or macrophage activation syndrome. *Lancet Rheumatol.* 2, e358-e367. 10.1016/S2665-9913(20)30096-5 32373790 PMC7198216

[DMM050762C43] Mehta, P., Mcauley, D. F., Brown, M., Sanchez, E., Tattersall, R. S., Manson, J. J. and Hlh A. C.R.O.S.S. Speciality Collaboration, U. K. (2020b). COVID-19: consider cytokine storm syndromes and immunosuppression. *Lancet* 395, 1033-1034. 10.1016/S0140-6736(20)30628-0 32192578 PMC7270045

[DMM050762C44] Miettunen, P. M., Narendran, A., Jayanthan, A., Behrens, E. M. and Cron, R. Q. (2011). Successful treatment of severe paediatric rheumatic disease-associated macrophage activation syndrome with interleukin-1 inhibition following conventional immunosuppressive therapy: case series with 12 patients. *Rheumatology (Oxf.)* 50, 417-419. 10.1093/rheumatology/keq218 20693540

[DMM050762C45] Nakamura, K., Okamura, H., Wada, M., Nagata, K. and Tamura, T. (1989). Endotoxin-induced serum factor that stimulates gamma interferon production. *Infect. Immun.* 57, 590-595. 10.1128/iai.57.2.590-595.1989 2492265 PMC313137

[DMM050762C46] Naymagon, L. (2022). Anakinra for the treatment of adult secondary HLH: a retrospective experience. *Int. J. Hematol.* 116, 947-955. 10.1007/s12185-022-03430-9 35948764 PMC9365216

[DMM050762C47] Novick, D., Kim, S. H., Fantuzzi, G., Reznikov, L. L., Dinarello, C. A. and Rubinstein, M. (1999). Interleukin-18 binding protein: a novel modulator of the Th1 cytokine response. *Immunity* 10, 127-136. 10.1016/S1074-7613(00)80013-8 10023777

[DMM050762C48] Okamura, H., Tsutsi, H., Komatsu, T., Yutsudo, M., Hakura, A., Tanimoto, T., Torigoe, K., Okura, T., Nukada, Y., Hattori, K. et al. (1995). Cloning of a new cytokine that induces IFN-γ production by T cells. *Nature* 378, 88-91. 10.1038/378088a0 7477296

[DMM050762C49] Phadke, O., Rouster-Stevens, K., Giannopoulos, H., Chandrakasan, S. and Prahalad, S. (2021). Intravenous administration of anakinra in children with macrophage activation syndrome. *Pediatr. Rheumatol. Online J.* 19, 98. 10.1186/s12969-021-00585-3 34187503 PMC8240425

[DMM050762C50] Ragab, D., Salah Eldin, H., Taeimah, M., Khattab, R. and Salem, R. (2020). The COVID-19 cytokine storm; what we know so far. *Front. Immunol.* 11, 1446. 10.3389/fimmu.2020.01446 32612617 PMC7308649

[DMM050762C51] Ravelli, A., Minoia, F., Davi, S., Horne, A., Bovis, F., Pistorio, A., Arico, M., Avcin, T., Behrens, E. M., De Benedetti, F. et al. (2016). 2016 classification criteria for macrophage activation syndrome complicating systemic juvenile idiopathic arthritis: a European league against rheumatism/American College of Rheumatology/Paediatric Rheumatology International Trials Organisation Collaborative Initiative. *Arthritis Rheumatol.* 68, 566-576. 10.1002/art.39332 26314788

[DMM050762C52] Sandler, R. D., Tattersall, R. S., Schoemans, H., Greco, R., Badoglio, M., Labopin, M., Alexander, T., Kirgizov, K., Rovira, M., Saif, M. et al. (2020). Diagnosis and management of secondary HLH/MAS following HSCT and CAR-t cell therapy in adults; a review of the literature and a survey of practice within EBMT Centres on Behalf of the Autoimmune Diseases Working Party (ADWP) and Transplant Complications Working Party (TCWP). *Front. Immunol.* 11, 524. 10.3389/fimmu.2020.00524 32296434 PMC7137396

[DMM050762C53] Schroder, K. and Tschopp, J. (2010). The inflammasomes. *Cell* 140, 821-832. 10.1016/j.cell.2010.01.040 20303873

[DMM050762C54] Schulert, G. S. and Cron, R. Q. (2020). The genetics of macrophage activation syndrome. *Genes Immun.* 21, 169-181. 10.1038/s41435-020-0098-4 32291394

[DMM050762C55] Seckinger, P., Lowenthal, J. W., Williamson, K., Dayer, J. M. and Macdonald, H. R. (1987). A urine inhibitor of interleukin 1 activity that blocks ligand binding. *J. Immunol.* 139, 1546-1549. 10.4049/jimmunol.139.5.1546 2957429

[DMM050762C56] Sepulveda, F. E., Debeurme, F., Menasche, G., Kurowska, M., Cote, M., Pachlopnik Schmid, J., Fischer, A. and De Saint Basile, G. (2013). Distinct severity of HLH in both human and murine mutants with complete loss of cytotoxic effector PRF1, RAB27A, and STX11. *Blood* 121, 595-603. 10.1182/blood-2012-07-440339 23160464 PMC3824285

[DMM050762C57] Shakoory, B., Carcillo, J. A., Chatham, W. W., Amdur, R. L., Zhao, H., Dinarello, C. A., Cron, R. Q. and Opal, S. M. (2016). Interleukin-1 receptor blockade is associated with reduced mortality in sepsis patients with features of macrophage activation syndrome: reanalysis of a prior phase III trial. *Crit. Care Med.* 44, 275-281. 10.1097/CCM.0000000000001402 26584195 PMC5378312

[DMM050762C58] Shiga, T., Nozaki, Y., Tomita, D., Kishimoto, K., Hirooka, Y., Kinoshita, K., Funauchi, M. and Matsumura, I. (2021). Usefulness of interleukin-18 as a diagnostic biomarker to differentiate adult-onset still's disease with/without macrophage activation syndrome from other secondary hemophagocytic lymphohistiocytosis in adults. *Front. Immunol.* 12, 750114. 10.3389/fimmu.2021.750114 34691064 PMC8533049

[DMM050762C59] Shimizu, M., Yokoyama, T., Yamada, K., Kaneda, H., Wada, H., Wada, T., Toma, T., Ohta, K., Kasahara, Y. and Yachie, A. (2010). Distinct cytokine profiles of systemic-onset juvenile idiopathic arthritis-associated macrophage activation syndrome with particular emphasis on the role of interleukin-18 in its pathogenesis. *Rheumatology (Oxf.)* 49, 1645-1653. 10.1093/rheumatology/keq133 20472718

[DMM050762C60] Shimizu, M., Nakagishi, Y. and Yachie, A. (2013). Distinct subsets of patients with systemic juvenile idiopathic arthritis based on their cytokine profiles. *Cytokine* 61, 345-348. 10.1016/j.cyto.2012.11.025 23276493

[DMM050762C61] Sun, M., Brady, R. D., Wright, D. K., Kim, H. A., Zhang, S. R., Sobey, C. G., Johnstone, M. R., O'brien, T. J., Semple, B. D., Mcdonald, S. J. et al. (2017). Treatment with an interleukin-1 receptor antagonist mitigates neuroinflammation and brain damage after polytrauma. *Brain Behav. Immun.* 66, 359-371. 10.1016/j.bbi.2017.08.005 28782716

[DMM050762C62] Takada, H., Ohga, S., Mizuno, Y., Suminoe, A., Matsuzaki, A., Ihara, K., Kinukawa, N., Ohshima, K., Kohno, K., Kurimoto, M. et al. (1999). Oversecretion of IL-18 in haemophagocytic lymphohistiocytosis: a novel marker of disease activity. *Br. J. Haematol.* 106, 182-189. 10.1046/j.1365-2141.1999.01504.x 10444185

[DMM050762C63] Tone, M., Thompson, S. A., Tone, Y., Fairchild, P. J. and Waldmann, H. (1997). Regulation of IL-18 (IFN-gamma-inducing factor) gene expression. *J. Immunol.* 159, 6156-6163. 10.4049/jimmunol.159.12.6156 9550417

[DMM050762C64] Turnquist, C., Ryan, B. M., Horikawa, I., Harris, B. T. and Harris, C. C. (2020). Cytokine storms in cancer and COVID-19. *Cancer Cell* 38, 598-601. 10.1016/j.ccell.2020.09.019 33038939 PMC7531591

[DMM050762C65] Van De Veerdonk, F. L., Netea, M. G., Dinarello, C. A. and Joosten, L. A. (2011). Inflammasome activation and IL-1beta and IL-18 processing during infection. *Trends Immunol.* 32, 110-116. 10.1016/j.it.2011.01.003 21333600

[DMM050762C66] Vora, S. M., Lieberman, J. and Wu, H. (2021). Inflammasome activation at the crux of severe COVID-19. *Nat. Rev. Immunol.* 21, 694-703. 10.1038/s41577-021-00588-x 34373622 PMC8351223

[DMM050762C67] Wannamaker, W., Davies, R., Namchuk, M., Pollard, J., Ford, P., Ku, G., Decker, C., Charifson, P., Weber, P., Germann, U. A. et al. (2007). (S)-1-((S)-2-[1-(4-amino-3-chloro-phenyl)-methanoyl]-amino-3,3-dimethyl-butanoyl)-pyrrolidine-2-carboxylic acid ((2R,3S)-2-ethoxy-5-oxo-tetrahydro-furan-3-yl)-amide (VX-765), an orally available selective interleukin (IL)-converting enzyme/caspase-1 inhibitor, exhibits potent anti-inflammatory activities by inhibiting the release of IL-1beta and IL-18. *J. Pharmacol. Exp. Ther.* 321, 509-516. 10.1124/jpet.106.111344 17289835

[DMM050762C68] Weiss, E. S., Girard-Guyonvarc'h, C., Holzinger, D., De Jesus, A. A., Tariq, Z., Picarsic, J., Schiffrin, E. J., Foell, D., Grom, A. A., Ammann, S. et al. (2018). Interleukin-18 diagnostically distinguishes and pathogenically promotes human and murine macrophage activation syndrome. *Blood* 131, 1442-1455. 10.1182/blood-2017-12-820852 29326099 PMC5877443

[DMM050762C69] Yasin, S., Fall, N., Brown, R. A., Henderlight, M., Canna, S. W., Girard-Guyonvarc'h, C., Gabay, C., Grom, A. A. and Schulert, G. S. (2020a). IL-18 as a biomarker linking systemic juvenile idiopathic arthritis and macrophage activation syndrome. *Rheumatology (Oxf.)* 59, 361-366. 10.1093/rheumatology/kez282 PMC7571484 31326996

[DMM050762C70] Yasin, S., Solomon, K., Canna, S. W., Girard-Guyonvarc'h, C., Gabay, C., Schiffrin, E., Sleight, A., Grom, A. A. and Schulert, G. S. (2020b). IL-18 as therapeutic target in a patient with resistant systemic juvenile idiopathic arthritis and recurrent macrophage activation syndrome. *Rheumatology (Oxf.)* 59, 442-445. 10.1093/rheumatology/kez284 PMC8453620 31359055

