# Supplementary material for: The NLRP3 inflammasome is essential for IL-18 production in a murine model of macrophage activation syndrome
Source: Dis Model Mech. 2024 Jul 30;17(7):dmm050762. doi: 10.1242/dmm.050762 (PMC11317095; doi:10.1242/dmm.050762)
Supplement: Supplementary information [file dmm-17-050762-s1.pdf]

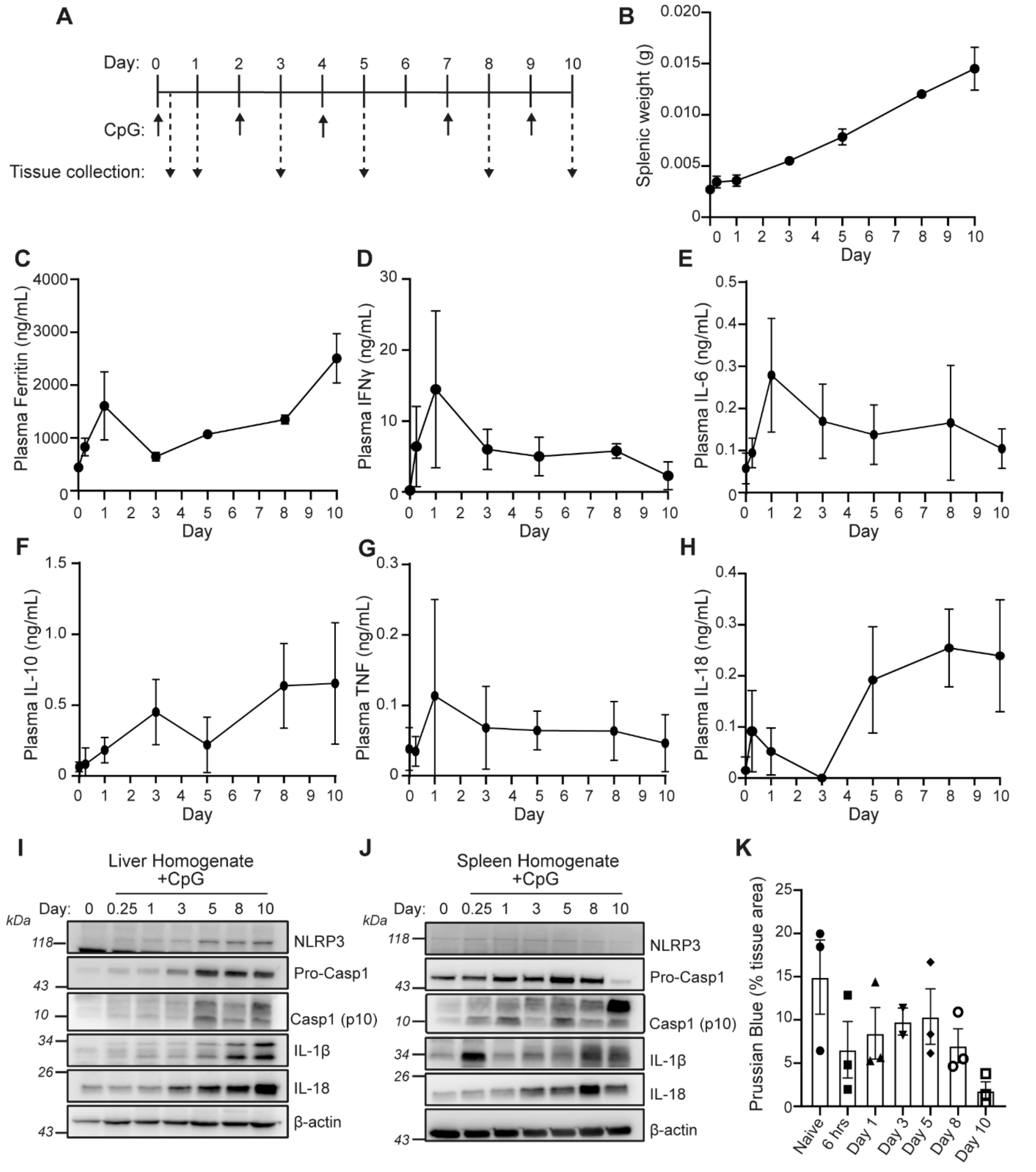

Fig. S1. The upregulation of Inflammasome signalling requires multiple doses of CpG-DNA. (A) To examine the kinetics disease progression in CpG-induced MAS, tissues were taken at different timepoints in the development of MAS. Mice received either a single dose of CpG (2 mg kg^{-1}) with tissue collected 6 h or 24 h post-injection, or cumulative doses of CpG on day 0, 2, 4, 7, and 9, with tissue collected from discrete groups 24 h after each injection (on day 3, 5, 8 and 10 respectively). Naïve mice were used to determine baseline (day 0). **(B)** Splenic weight normalised to body weight over time in mice treated as in (A) (n=3). **(C-H)** Plasma levels of ferritin (C), IFNγ (D), IL-6 (E), IL-10 (F), TNF (G) and IL-18 (H) over time in mice treated as in (A) (n=3). **(I-J)** Western blot analysis of liver (I) and spleen (J) homogenate from mice treated as in (A) of the inflammasome components NLRP3, pro-caspase-1, cleaved caspase-1 (p10), IL-18 and IL-1β (n=3) **(K)** Iron staining of liver over time in mice treated with CpG-DNA. Prussian blue staining was quantified in Qupath and staining is expressed as a percentage of total tissue area (n=3, apart from day 3, where n=2). Values shown are the mean ±SEM.

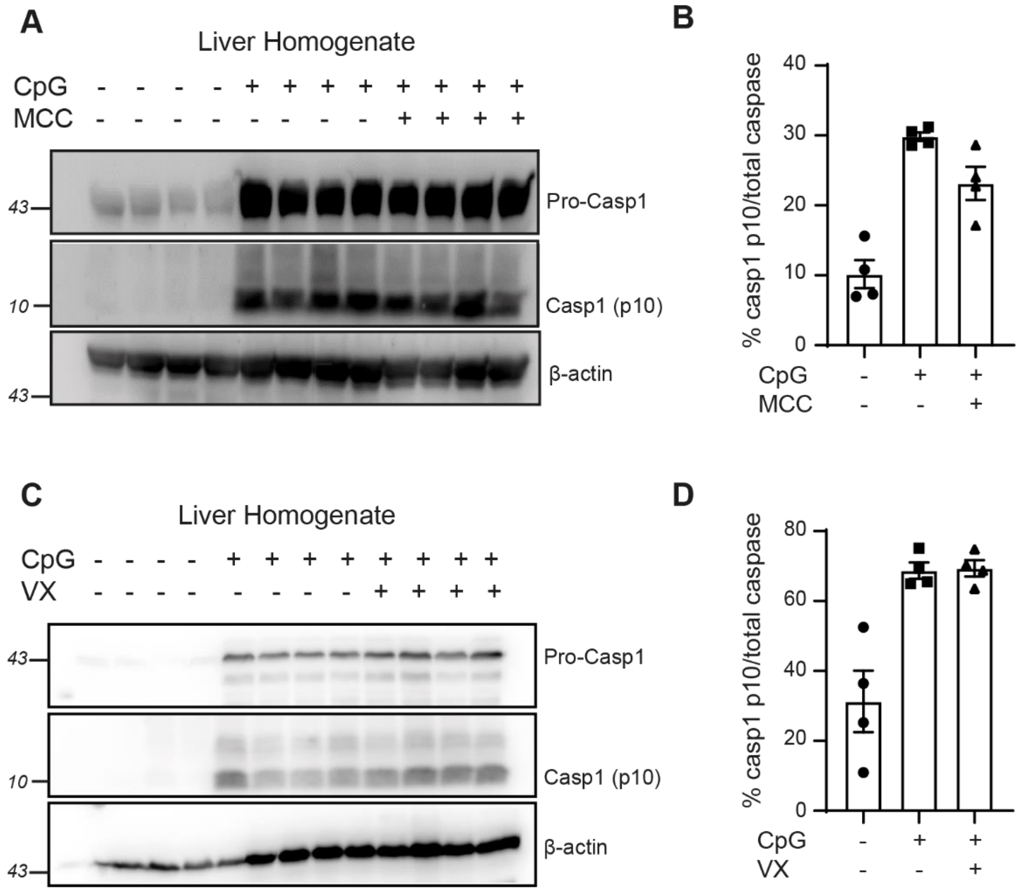

Fig. S2. Inhibition of the NLRP3 inflammasome and caspase-1 did not prevent pro-caspase-1 cleavage in the liver of mice treated with repeated doses of CpG-DNA. (A) Western blot of homogenised livers from PBS/PBS, CpG/PBS or CpG/MCC950 treated animals were blotted for pro-caspase-1 and the active caspase-1 p10 subunit (n=4). **(C)** Western blot of homogenised livers from PBS/Veh, CpG/Veh or CpG/VX765 treated animals were blotted for pro-caspase-1 and the active caspase-1 p10 subunit (n=4). **(B, D)** Bar graphs show densitometry data where cleaved caspase-1 (p10) is expressed as a percentage of total caspase-1, corresponding to MCC950 study (B) and VX765 study (D), respectively (n=4). Values shown are mean ±SEM.

Disease Models & Mechanisms • Supplementary information
